# Dyke-Davidoff-Masson Syndrome: A Case Report and Review of Literature

**DOI:** 10.7759/cureus.11919

**Published:** 2020-12-05

**Authors:** Amna Younas, Muhammad Saim, Hamza Maqsood, Shifa Younus, Muhammad Hassan Raza

**Affiliations:** 1 Medicine, Russells Hall Hospital, Dudley, GBR; 2 Department of Cardiology, Nishtar Medical University, Multan, PAK; 3 Department of Cardiovascular Medicine, Nishtar Medical University, Multan, PAK; 4 Department of Radiology, Nishtar Medical University, Multan, PAK; 5 Department of Internal Medicine, Nishtar Medical University, Multan, PAK

**Keywords:** cerebral atrophy, hemiplegia, seizures, ddms

## Abstract

Dyke-Davidoff-Masson syndrome (DDMS) is a rare neurological disorder that results from brain injury in intrauterine or early years of life. Prominent cortical sulci, dilated lateral ventricles, cerebral hemiatrophy, hyperpneumatization of the frontal sinus, and compensatory hypertrophy of the skull are the characteristic findings. We describe a male patient who presented with generalized tonic-clonic seizure and left-sided body weakness and neuroimaging findings of cerebral hemiatrophy, dilatation of right lateral ventricle, right frontal sinus hyperpneumatization, and asymmetric calvarial thickening. Knowledge of its features on imaging enables timely and accurate diagnosis, allowing appropriate management.

## Introduction

Cerebral hemiatrophy or Dyke-Davidoff-Masson syndrome (DDMS) is a rare neurological condition that was first described by Dyke, Davidoff, and Masson in a series of nine patients with hemiplegia and plain skull X-ray changes [1,2]. It is characterized by cerebral hemiatrophy/hypoplasia, facial asymmetry, seizures, and contralateral hemiplegia [1-3]. These clinical features can present with diverse combinations and severity. Imaging studies are utilized to make a diagnosis in correlation with clinical features. Specific imaging findings include unilateral brain volume loss, ventriculomegaly, and compensatory bone hypertrophy resulting in cerebral hemiatrophy. In addition, calvarial thickening and hyperpneumatization of frontal sinuses may occur [3,4]. As it is a rare disorder, it may be misdiagnosed and consequently mismanaged by the majority of physicians.

## Case presentation

Here we report a case of a 13-years-old boy who presented in a medical emergency with complaints of generalized tonic-clonic seizures and right hemiparesis. The hemiparesis was non-progressive and started at the age of ten years. He also had some behavioral changes that started at the age of six years, along with frequent episodes of mild headache. The behavioral changes included disturbed sleep, irrelevant talks, and irritability. There was no history of similar presentations in any other sibling or family member.

On general physical examination, there was emaciation with decreased cognitive functioning; 13/30 on the Mini-Mental State Examination. On neurological examination, the power was 3/5 on the left side of the body with decreased sensations, hypertonia, and brisk reflexes. We also observed mild left-sided facial angle deviation. The rest of the systemic examination was unremarkable. Laboratory workup included baseline investigations and a thorough workup for young stroke along with an autoimmune profile. All laboratory investigations were within a normal range.

**Figure 1:**
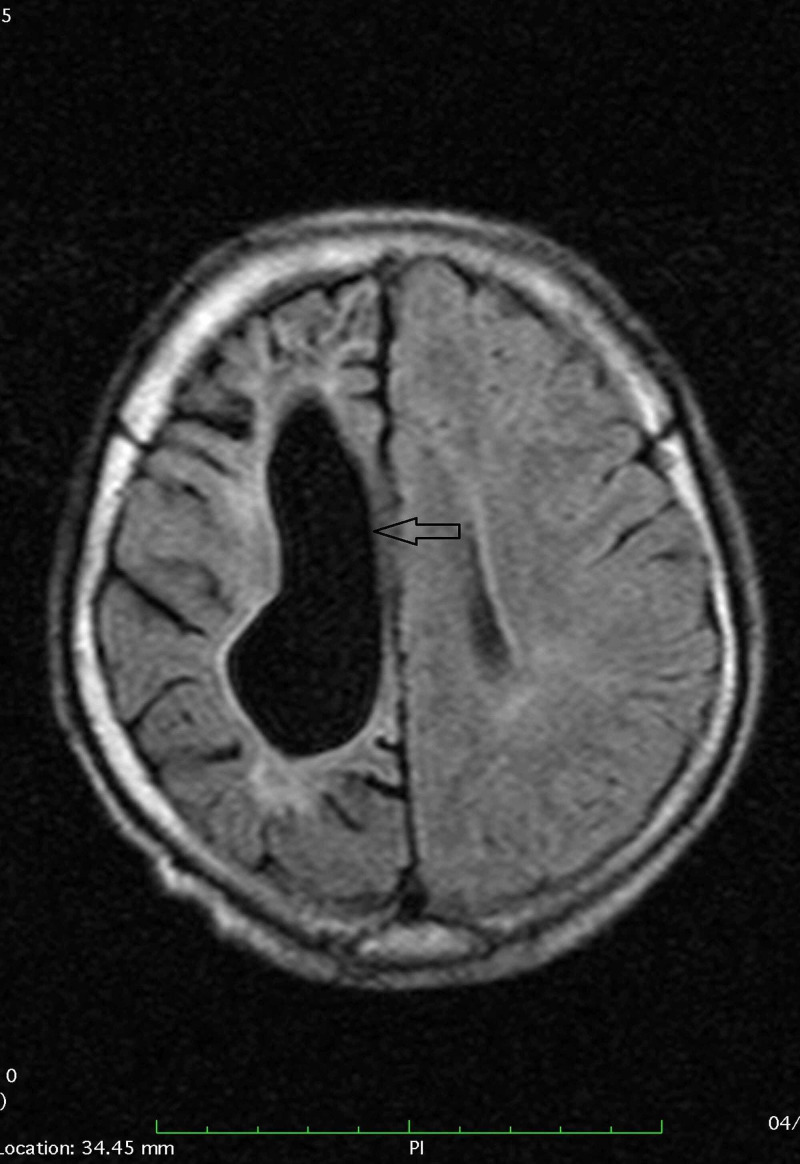
CT scan image showing the atrophy of the right cerebral hemisphere with the prominence of extra-axial cerebrospinal fluid (CSF) spaces (black arrow)

We did a CT scan brain without contrast, which revealed atrophy of the right cerebral hemisphere, with the prominence of extra-axial cerebrospinal fluid (CSF) spaces and dilatation of the ipsilateral right lateral ventricle. It also revealed low attenuation areas in the right frontal and right parietal lobes depicting possible subacute infarct in the right frontal and acute infarct in the right parietal region (Figure 1).

**Figure 2:**
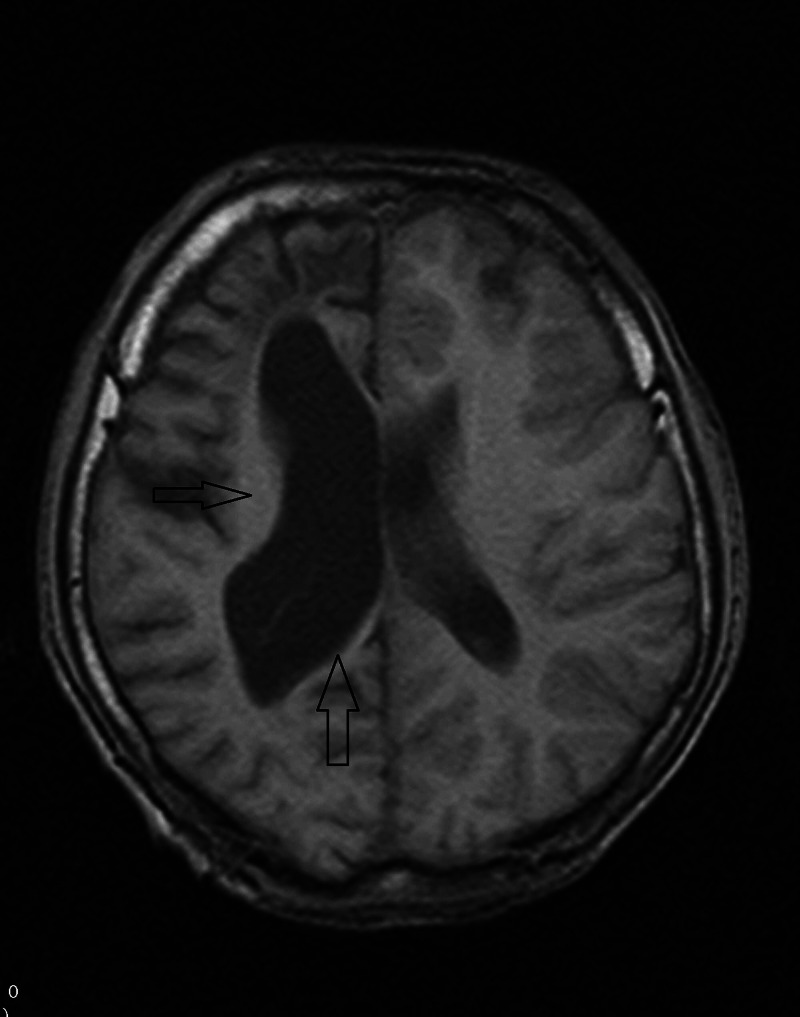
MRI (T1W) showing atrophic right cerebral hemisphere with dilatation of the right lateral ventricle and extra-axial cerebrospinal fluid (CSF) spaces (black arrows)

We also did magnetic resonance imaging (MRI), which revealed an atrophic right cerebral hemisphere, with dilatation of the right lateral ventricle and extra-axial CSF spaces with mild asymmetry in the calvarial thickness of both hemispheres (Figure 2).

**Figure 3:**
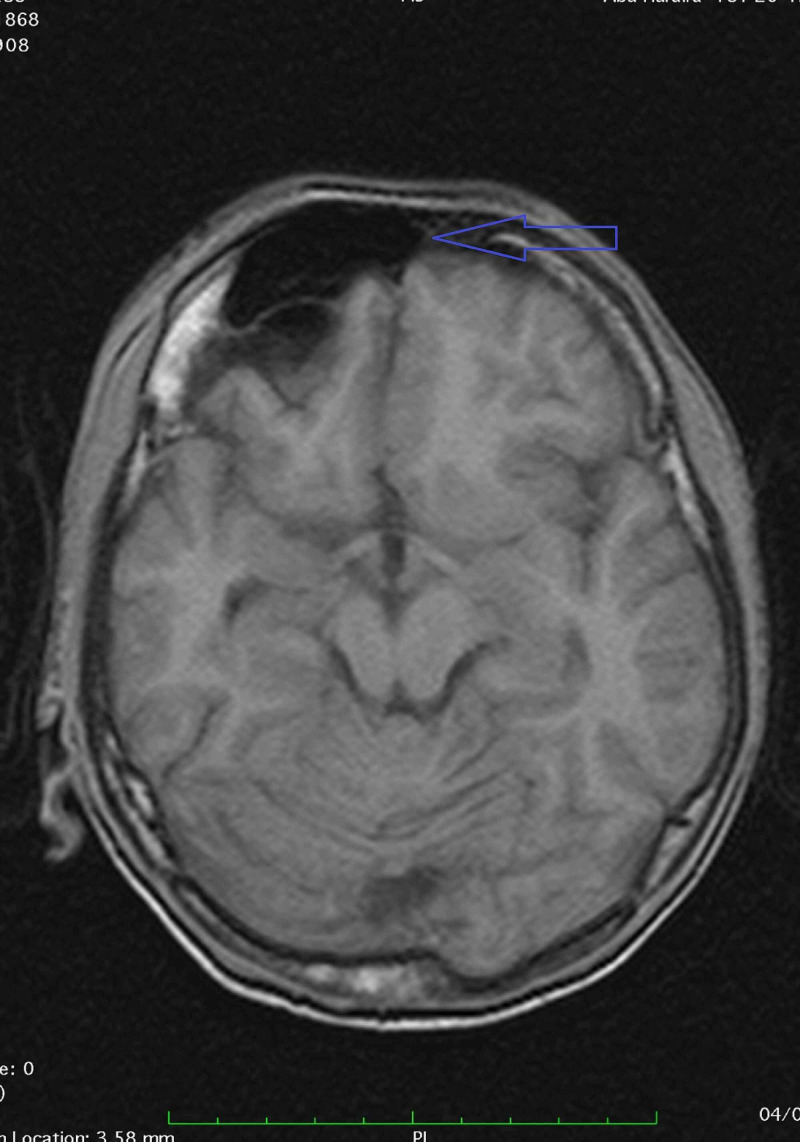
MRI showing hyperpneumatization of right frontal sinus (blue arrow) as compared to the left one

MRI also showed a prominent right frontal sinus as compared to the left one depicting hyperpneumatization. We also observed various areas of encephalomalacia with surrounding gliosis in the left frontal region that is suggestive of previous brain insult (Figure 3).

After correlating the clinical presentation and imaging findings, a final diagnosis of DDMS was made. His treatment regimen consisted of anti-epileptic medications and physiotherapy. We referred him to neurology inpatient services for targeted interventions and management.

## Discussion

This rare syndrome was first described by Dyke, Davidoff, and Masson back in 1933. They observed that a few patients presented with a specific cluster of symptoms including hemiparesis, facial asymmetry, seizures, and mental retardation. All those patients showed classical unilateral cerebral atrophy on skull radiography [5]. Predominantly, there is no established sex predilection or involvement of a specific hemisphere but, the involvement of the left side and male gender are more common in the literature [6].

The clinical presentation of DDMS include seizures, contralateral hemiparesis of upper motor neuron type disease, facial asymmetry, and cognitive disabilities. DDMS can be classified into two forms depending upon its etiology. The congenital subtype, which becomes symptomatic in infancy, and its pathogenesis include fetal vascular occlusion. The other is the acquired subtype, which presents in childhood. Its etiological factors include perinatal hypoxia, intracranial hemorrhage, infections, cranial trauma, and cerebrovascular lesion [7]. The possible mechanism of cerebral atrophy and the related progressive neuro deficit is hypothesized to be due to several ischemic episodes resulting from these factors, which reduce the production of brain-derived neurotrophic factors, which in turn leads to cerebral atrophy [8].

CT and MRI are the two gold standard imaging modalities that prove to be very significant in the diagnosis of DDMS. These two imaging modalities provide very detailed cross-sectional images. The typical imaging features for DDMS include prominent cortical sulci, dilated lateral ventricles, cerebral hemiatrophy, hyperpneumatization of the frontal sinus, and compensatory hypertrophy of the skull. These imaging findings become more obvious as the patient ages [9]. When the cerebral damage occurs during the intrauterine period or before the age of 3, compensatory calvarial involvement can be seen [10,11].

In a patient with cerebral hemiatrophy, the differential diagnosis includes Rasmussen encephalitis, Sturge-Weber syndrome, basal ganglia dysgerminoma, Fisherman syndrome, and Silver-Russell syndrome. Detailed history and examination are required along with laboratory and imaging workup to differentiate these diseases.

For the management of seizures, mono, or poly anticonvulsant medication is given. Children with refractory epilepsy and hemiplegia are potential candidates for hemispherectomy, with a success rate of 85%. Vagal stimulation is another alternative. Despite lacking any specific treatment algorithm, therapy with antiepileptics and surgery is indicated in specific cases. Long-term supportive management includes physical, language, and occupational therapy [4].

## Conclusions

DDMS is a rare neurological disorder leading to intractable seizures along a spectrum of disabilities. As it is a rare syndrome, it may be easily misdiagnosed by the inexperienced eye. Detailed history and examination are needed with imaging modalities to diagnose it. Physicians should be aware of signs and symptoms, risk factors, and diagnostic features of DDMS so that the patients could be managed properly.
